# A26 BIOLOGICAL CHARACTERIZATION OF THE INTERACTION BETWEEN SIRT1 AND HNF4Α2 IN INTESTINAL EPITHELIAL CELLS

**DOI:** 10.1093/jcag/gwad061.026

**Published:** 2024-02-14

**Authors:** J A Herrera Pulido, C Jones, F Boudreau

**Affiliations:** . Immunology and cell biology, Universite de Sherbrooke, Sherbrooke, QC, Canada; . Immunology and cell biology, Universite de Sherbrooke, Sherbrooke, QC, Canada; . Universite de Sherbrooke, Sherbrooke, QC, Canada

## Abstract

**Background:**

Colorectal cancer (CRC) is the third most common cancer in Canada. *HNF4A* locus is amplified in CRC, while additional reports suggest that hepatocyte nuclear factor 4 alpha (HNF4α) is associated with increased proliferation and disease development. This dual role as a tumor suppressor or oncoprotein might be due to the production of 12 spliced isoforms with structural differences and variable tissue expression. It suggests distinct functions for each isoform according to their specific interaction complexes. However, little is known about the nature of these protein complexes and their biological functions during intestinal physiopathology. Recently, using a BioID2 and mass spectrometry approach, our laboratory showed a possible interaction between HNF4α2 and sirtuin 1 (SIRT1) in intestinal epithelial cells from a colon carcinoma (HCT116). HNF4α2 is one of the most potent isoforms in the control of transcriptional activity of multiple intestinal epithelial genes related to regulating cell death, angiogenesis, apoptosis, response to injury, and response to drugs, among others.

**Aims:**

To characterize the possible interaction between HNF4α2 and SIRT1 in intestinal epithelial cells at the biological level.

**Methods:**

We performed SIRT1 knockdown using shRNAs in HCT116 cells expressing the HNF4α2 isoform in an inducible manner by doxycycline (DOX). Subsequently, we conducted a proximity ligation assay to validate the interaction between HNF4α2 and SIRT1 in HCT116 cells. Additionally, protein purification was performed to validate their physical interaction through EMSA. A transcriptome analysis was carried out using RNA-seq to define the biological processes involved in the interaction between the two proteins.

**Results:**

We observed an interaction signal between HNF4α2 and SIRT1 in HCT116 cells, similar to that observed for IRFBB2, whose interaction with HNF4α2 has been previously validated. This signal decreased when cells were treated with shRNA for SIRT1 and was not observed in cells not induced by DOX. EMSA analyses revealed a direct interaction signal between HNF4α2 and SIRT1. RNA-seq analyses showed a loss in the regulation of over 95% of genes targeted by HNF4α2 when cells were treated with shRNA for SIRT1. An enrichment analysis for GO annotations using ShyniGO v. 0.77 software showed an enrichment of genes for biological processes such as apoptosis, inflammation, cell migration, cell invasion, cell death, and various cancer-related signaling pathways.

**Conclusions:**

SIRT1 displays physical interaction with HNF4α2 and significantly affects the transcriptional regulation of this isoform in the context of epithelial cells. Their interaction is involved in processes related to intestinal inflammation and cancer progression. Pharmacological targeting of SIRT1 could represent a viable strategy in treating CRC and other intestinal diseases

**Funding Agencies:**

CIHRNSERC

